# A Novel Two-Step Ultrasound-Guided Technique for Anesthesia and Postsurgery Analgesia in Gluteal Augmentation Surgery

**DOI:** 10.7759/cureus.43546

**Published:** 2023-08-15

**Authors:** Mario Fajardo Perez, Karla Espinoza Morales, Jose Fabio Rodriguez Sojo, Yaneth Prada Castellnos, Felice Galluccio

**Affiliations:** 1 Pain Medicine, Morphological Madrid Research Center (MoMaRC), Madrid, ESP; 2 Anatomical Lab, Universidad Cientifica del Sur, Lima, PER; 3 Anesthesiology, Hospital Centro de Atención Integral en Salud (CAIS) de Puriscal, San José, CRI; 4 Anesthesia, Centro Europeo de Cirugía, La Sabana, CRI; 5 Plastic Surgery, Hospital San Juan de Dios, San José, CRI; 6 Rheumatology & Rehabilitation, Fisiotech Lab Studio, Firenze, ITA

**Keywords:** botox injections, gluteal augmentation surgery, gluteoplasty, locoregional anesthesia, tumescent anesthesia

## Abstract

In recent years, there has been an increase in demand for gluteal augmentation and reshaping surgeries with intramuscular implants which are becoming increasingly popular. Until now, this surgery was mainly performed under general anesthesia, but recently locoregional anesthesia techniques, such as tumescent local anesthesia, are being applied more and more. Today, the use of ultrasound for locoregional anesthesia and analgesia allows us to perform techniques that are increasingly precise and burdened with lower risks. In this report, we present a novel two-step ultrasound-guided technique combining a botox injection in the gluteus maximus four weeks before surgery and tumescent anesthesia with a cluneal nerve block. Furthermore, the combination of anesthetic and analgesic techniques can guarantee a better result both in terms of surgical execution of the intervention and in reducing pain and improving patient comfort in the postoperative period.

## Introduction

In recent years, there has been an increase in the demand for gluteal augmentation and remodeling surgery. Among the various surgical options, in addition to liposuction and liposculpture with fat harvesting and grafting, the placement of intramuscular implants is becoming more and more popular [1,2]. Intramuscular implants, compared to subcutaneous or subfascial and submuscular ones, guarantee a more natural effect both in terms of palpability and visibility, and this technique can be combined with autologous fat grafting to obtain the best possible appearance. Furthermore, intra and submuscular implantation techniques have the advantage of having a lower complication rate, especially implant malposition, extrusion, or displacement [3]. In gluteoplasty with intra or submuscular implants, postoperative pain due to contracture of the gluteus maximus muscle is one of the main complaints made by patients. This may be due to various factors, including degeneration of muscle fibers with glycogen deposits and interstitial fibrosis, attributed to tissue hypoxia secondary to muscle spasm [4-6]. 

Gluteal augmentation surgery can be performed under both local and general anesthesia, depending on the surgical needs and specific conditions of the case. Among the locoregional anesthesia techniques, a recent article on tumescent local anesthesia (TLA), which consists of the infiltration of large volumes of physiological solution with lidocaine and epinephrine into the muscle compartment, has been recently published, with good results [7]. 

To avoid painful contracture of the gluteus maximus muscle and thus improve the postoperative experience, we have studied and are applying an anesthetic and analgesic alternative that helps improve the comfort and outcome of patients undergoing this surgical procedure and involves pretreatment of the gluteus maximus with botulinum toxin followed by an ultrasound-guided TLA and cluneal nerve block the day of the surgery. 

## Technical report

**Figure 1:**
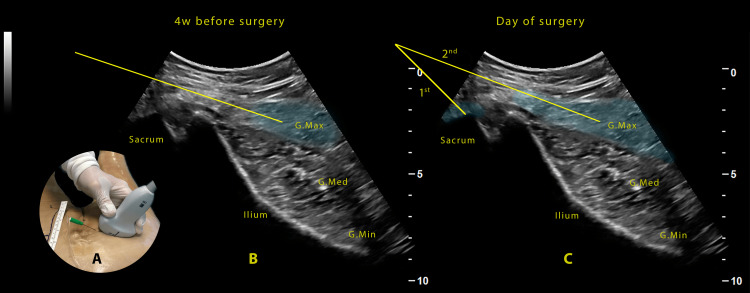
Ulrasound-guided procedure. Proposed ultrasound-guided technique for gluteal anesthesia and analgesia. (A) Probe and needle position. (B) Botox infiltration technique for gluteus maximus four weeks before planned surgery. (C)  Ultrasound-guided infiltration of the middle cluneal nerves (1st) and tumescent solution infiltration of the gluteus maximus (2nd). G.max: gluteus maximus; G.Med: Gluyeus medium; G.Min: gluteus minimus

We propose performing an ultrasound-guided infiltration of botulinum toxin (btx - Botox Allergan Pharmaceuticals Ireland®) in the gluteus maximus muscle four weeks before the surgical procedure, after which, as reported by surgeons, the gluteus maximus muscle will present in better conditions for intraoperative manipulation, with more relaxation and less contractility or spasms. The suggested btx dose is 100 units per side. To scan the gluteus, the ultrasound probe is placed over the blade of the ilium, identified as a hyperechoic line with the three gluteal muscles resting above (maximus, medium, and minimus). The needle is inserted, medial to lateral and in-plane, through the gluteus maximus muscle (Figure 1A). The btx infiltration will be performed during needle retraction, spreading the toxin throughout the muscle belly (Figure 1B). It is fundamental to avoid the injection near the superior gluteal artery, which can be easily visualized between the maximus and medium gluteus, to avoid the block of the superior gluteal nerve, since it innervates the gluteus medius muscle, the major stabilizer of the hip, because it could temporarily compromise walking, delaying discharge and increasing the risk of falling in the perioperative period. On the day of the surgery, ultrasound-guided infiltration of the middle cluneal nerves with a local anesthetic will be performed at the level of the lateral edge of the sacrum and the gluteus maximus muscle with the tumescent solution. The middle cluneal nerves originate from the posterior rami of the first to third sacral nerves (S1-S3). These nerves run throughout the skin of the gluteal region, innervating the skin over the gluteus maximus muscle, where the surgical incision is made [8]. To inject the cluneal nerves, with the patient in the prone position and after adequate skin disinfection, we proceed with the ultrasound scan as before, with a linear or convex probe, placing the probe at the sacrum border. Once identified the correct position and after a subcutaneous lidocaine 1% 1-2cc injection (lidocaine; B.Braun Inc., USA®), the needle was inserted, in-plane or out-of-plane as preferred, until the tip touches the lateral crest of the sacrum, and 5ml of the anesthetic solution is injected (Figure 1C). It is possible that a part of the injectate may reach the lateral sacral branches, but generally negligible.

Then it is possible to redirect the needle through the gluteus maximus muscle and inject the tumescent solution (Figure 1C). We propose a tumescent solution as follows: in 250 cc of saline, add 200 mg of 0.5% bupivacaine or levobupivacaine, 8 mEq of sodium bicarbonate, and 1 mg of epinephrine (1 cc), resulting in a concentration of epinephrine of 4mcg/ml, injecting 20ml of this solution. The solution should be injected into the gluteus maximus muscle approximately 20-30 minutes before surgery to achieve the anesthetic effect.

## Discussion

Among the advantages of performing the ultrasound-guided technique are the precision and safety of the injection. It decreases the possibility of accidental vascular puncture of vein branches and superior and inferior gluteal arteries compared with blind techniques, avoiding unnecessary bleeding that will lead to a better surgical field and reducing the risk of hematoma [3,9].

Within the patients we have performed this technique, the major advantages of the btx infiltration in the gluteus maximus are a better relaxation of the muscle belly with less tension of the gluteal fold for easier implant placement, less risk of dehiscence of the sutures, and less postoperative pain and muscle spasms, with reduction of opioid assumption and its complications (postoperative nausea and vomiting, urinary retention, and pruritus, among others). This could result in a reduction in hospital stay and related costs and an earlier return to normal daily life activities for the patient.

In addition to analgesia, tumescent infiltration also helps decrease surgical bleeding and prolongation of the absorption of the local anesthetics because of epinephrine. It is our opinion that the use of long-acting local anesthetics, such as bupivacaine or levobupivacaine, is preferable to lidocaine or other fast-acting agents. There is a proportional relationship between the amount of drug administered and the resulting peak of local anesthetic in plasma. The blood concentration of a local anesthetic is determined by the amount injected and the rate of absorption from the injection site. The gluteus maximus is a very bulky muscle with a large blood supply. In tissue with an acidic pH, the ionized form predominates over the basic or non-ionized form, so the penetration of local anesthetics into the tissues is lower. This effect is reduced by adding bicarbonate to the tumescent solution [10].

Although it is not a complex technique and the learning curve is quite rapid, the staff must have the correct ultrasound training to using proper technique. Applying it correctly, the risk of systemic toxicity from local anesthetics, while always possible, is very low as well as the risk of other surgery-related complications.

## Conclusions

The remarkable anesthetic effects, combined with the influence of Botox on the gluteus muscle, contribute to expedited surgery, enhanced postoperative pain relief,and a quicker recovery process, ultimately elevating patient comfort. By implementing this technique and ensuring meticulous preoperative planning, the intersurgical duration can be notably diminished, thereby augmenting the overall operational efficiency of clinics.
