# Correction: A Novel Two-Step Ultrasound-Guided Technique for Anesthesia and Postsurgery Analgesia in Gluteal Augmentation Surgery

**DOI:** 10.7759/cureus.c292

**Published:** 2025-09-16

**Authors:** Mario Fajardo Perez, Karla Espinoza Morales, Jose Fabio Rodriguez Sojo, Yaneth Prada Castellnos, Felice Galluccio

**Affiliations:** 1 Pain Medicine, Morphological Madrid Research Center (MoMaRC), Madrid, ESP; 2 Anatomical Lab, Universidad Cientifica del Sur, Lima, PER; 3 Anesthesiology, Hospital Centro de Atención Integral en Salud (CAIS) de Puriscal, San José, CRI; 4 Anesthesia, Centro Europeo de Cirugía, La Sabana, CRI; 5 Plastic Surgery, Hospital San Juan de Dios, San José, CRI; 6 Rheumatology & Rehabilitation, Fisiotech Lab Studio, Firenze, ITA

The Ethics Statement and Conflict of Interest Disclosures section has been updated to disclose the following:

**Financial relationships:** Jose Fabio Rodriguez Sojo declare(s) employment from Centro Europeo de Cirugía. Centro Europeo de Cirugía is a private, for-profit surgical clinic specializing in aesthetic, reconstructive, and plastic surgery. Felice Galluccio declare(s) employment from Fisiotech Lab Studio. Fisiotech Lab Studio is a private, for-profit medical facility offering rheumatology, pain therapy, and regenerative treatments.

